# The Potential Role of Matrix Metalloproteinases 8 and 9 and Myeloperoxidase in Predicting Outcomes of Bacterial Meningitis of Childhood

**DOI:** 10.1155/2019/7436932

**Published:** 2019-11-03

**Authors:** Okko Savonius, Irmeli Roine, Saeed Alassiri, Taina Tervahartiala, Otto Helve, Josefina Fernández, Heikki Peltola, Timo Sorsa, Tuula Pelkonen

**Affiliations:** ^1^Children's Hospital, Pediatric Research Center, University of Helsinki, Helsinki University Hospital, Helsinki, Finland; ^2^Faculty of Medicine, University Diego Portales, Santiago, Chile; ^3^Department of Oral and Maxillofacial Diseases, Institute of Dentistry, Helsinki University Hospital and the University of Helsinki, Helsinki, Finland; ^4^Clínica Infantil Dr. Robert Reid Cabral, Santo Domingo, Dominican Republic; ^5^Department of Dental Medicine, Karolinska Institutet, Huddinge, Sweden

## Abstract

**Background:**

Matrix metalloproteinases (MMPs) and myeloperoxidase (MPO) contribute to the inflammatory cascade in the cerebrospinal fluid (CSF) during bacterial meningitis. We determined levels of MPO, MMP-8, MMP-9, and tissue inhibitor of metalloproteinase- (TIMP-) 1 in the CSF of children with bacterial meningitis and investigated how these inflammatory mediators relate to each other and to the disease outcomes.

**Methods:**

Clinical data and the diagnostic CSF samples from 245 children (median age eight months) with bacterial meningitis were obtained from a clinical trial in Latin America in 1996–2003. MMP-9 levels in the CSF were assessed by zymography, while MMP-8, MPO, and TIMP-1 concentrations were determined with immunofluorometric and enzyme-linked immunosorbent assays.

**Results:**

MPO correlated positively with MMP-8 (rho 0.496, *P* < 0.001) and MMP-9 (rho 0.153, *P* = 0.02) but negatively with TIMP-1 (rho -0.361, *P* < 0.001). MMP-8 emerged as the best predictor of disease outcomes: a CSF MMP-8 concentration above the median increased the odds of death 4.9-fold (95% confidence interval 1.8–12.9).

**Conclusions:**

CSF MMP-8 presented as an attractive prognostic marker in children with bacterial meningitis.

## 1. Introduction

Bacterial meningitis (BM) remains a significant cause of childhood mortality and morbidity globally, often affecting children in developing, resource-poor countries [1]. Invading bacteria trigger a strong host reaction, which is observed in the cerebrospinal fluid (CSF) as the release of proinflammatory mediators [2]. The current understanding is that this intense proinflammatory cascade at least in part accounts for poor outcomes, so common in BM [2].

Matrix metalloproteinases (MMPs) are a structurally related but genetically distinct group of proteolytic enzymes which play a central role in regulating tissue destruction, remodeling, and immune responses, including in BM [3–5]. Within this proteinase group, marked differences exist in terms of expression: inductive MMPs are upregulated in inflammatory conditions, while others are rather consistently expressed [6, 7]. The activities of MMPs are further regulated by means of compartmentalization, as well as secretion as inactive zymogens requiring activation before being catalytically competent. Finally, active MMPs may be inhibited by tissue inhibitors of metalloproteinases (TIMPs), acting as their endogenous regulators [8].

The proinflammatory burden in the CSF during BM induces the production of reactive oxygen species, catalyzed by the enzyme myeloperoxidase (MPO), among others. Interestingly, in addition to targeting microbes, MPO is capable of both oxidatively activating latent pro-MMPs and inactivating TIMPs [9, 10]. Thus, MPO serves as a link between the oxidative burst and the proteolytic web of MMPs. In fact, the potential of reactive oxygen species to activate MMPs has been suggested a possible target for adjuvant treatment in BM [11].

In a previous study, our group showed that CSF MMP-9 is strongly upregulated in BM and that increased MMP-9 levels on admission associate with severe disease and an increased risk of death [12]. The release of MMP-8 is also upregulated in BM [13, 14], but no correlation with outcome has been found [13].

To our knowledge, however, no previous studies have explored the relation between these inflammatory mediators in a clinical setting. By measuring the MMP-8, MMP-9, TIMP-1, and MPO levels in the CSF of children with BM, we addressed two questions in this study: First, how would these inflammatory mediators relate to each other in human subjects? Second, to what extent would the results reflect the outcomes of this severe disease?

## 2. Materials and Methods

### 2.1. Patient Data

This study was a retrospective analysis using the prospectively collected data from a large double-blind treatment trial on childhood BM in Latin America in 1996–2003 [15]. The details of the study setup are described elsewhere [15], but in short, all children aged 2 months to 16 years received ceftriaxone for 7–10 days. In addition, the patients were randomized to receive either dexamethasone intravenously, glycerol orally, both agents, or only placebo as adjuvant treatment. CSF samples were collected on admission and after primary analyses frozen for later use. The study protocol was approved by all the local ethics committees. For this study, we included the patients from whom a frozen CSF sample was available.

On arrival at the hospital, the patients' clinical condition was graded using the age-adjusted Glasgow Coma Scale (GCS). Besides death, the disease outcomes were registered by defining as “severe neurological sequelae” all cases of blindness, quadriplegia, severe psychomotor retardation, or hydrocephalus requiring a shunt. “Any neurological sequelae” also comprised milder deficits such as ataxia and hemiparesis.

### 2.2. Immunofluorometry

Concentrations of MMP-8 were determined with a time-resolved immunofluorometric assay (Medix Biochemica, Espoo, Finland). The monoclonal MMP-8-specific antibodies 8708 and 8706 were used as a catching antibody and a tracer antibody, respectively. The tracer antibody was labeled using a europium chelate. The assay buffer contained 20 mM Tris-HCl, pH 7.5, 0.5 M NaCl, 5 mM CaCl_2_, 50 *μ*M ZnCl_2_, 0.5% bovine serum albumin, 0.05% sodium azide, and 20 mg/L diethylenetriaminepenta-acetic acid. Samples were diluted in assay buffer and incubated for 1 h, followed by incubation for 1 h with tracer antibody. Enhancement solution was added, and after 5 min, fluorescence was measured using a 1234 Delfia Fluorometer (Wallac, Turku, Finland). The interassay coefficient of variation was 7.3%, and the detection limit for the assay was 0.08 ng/mL.

### 2.3. Enzyme-Linked Immunosorbent Assays (ELISAs)

The levels of MPO and TIMP-1 were determined using commercially available ELISA kits. MPO ELISA (Immundiagnostik AG, Bensheim, Germany) and the Amersham Tissue Inhibitor of Metalloproteinases-1 (TIMP-1) Human Biotrak ELISA systems (Amersham Biosciences, GE Healthcare, Buckinghamshire, UK) were used according to the manufacturer's instructions. The secondary antibody in each kit was conjugated with horseradish peroxidase, and tetramethylbenzidine was used as a substrate. The absorbance was measured at 450 nm using a Victor X4 Multilabel Reader (PerkinElmer Finland Oy, Turku, Finland). The interassay coefficient of variation was <3% for MPO and <12% for TIMP-1, while the corresponding detection limits were 0.294 ng/mL and 1.25 ng/mL [16]. The levels of MMP-8 and TIMP-1 were expressed as ng/mL, and for calculation of the MMP-8/TIMP-1 molar ratios, the levels were converted to mol/L [17, 18].

### 2.4. Gelatin Zymography

The MMP-9 levels were assayed using zymography 11% sodium dodecyl sulphate-polyacrylamide gels, based on modification of the method of Lindberg et al. [14]. Our zymography gels were impregnated with 1 mg/mL gelatin as the substrate. Briefly, after 2 h preincubation with Laemmli's sample buffer without any reducing reagents, electrophoresis was performed. Thereafter, the gels were washed for 30 min twice with 50 mM Tris-HCl buffer solutions, pH 7.5, containing 2.5% Tween 80 and 0.02% NaN_3_; in addition, the second wash was supplemented with 0.5 mM CaCl_2_ and 1 *μ*M ZnCl_2_. Finally, the gels were incubated in 50 mM Tris-HCl buffer, pH 7.5, containing 0.02% NaN_3_, 0.5 mM CaCl_2_, and 1 *μ*M ZnCl_2_ but no Tween 80, overnight at 37°C. The gels were then stained with 1% Coomassie Brilliant Blue R-250, and the gelatinolytic activity was visualized as clear bands against the blue background on stained gels [19]. The intensities of gelatinolytic activity were evaluated with a Bio-Rad Model GS-700 Imaging Densitometer using the Bio-Rad Quantity One program (Bio-Rad Laboratories, Hercules, CA, USA) [14]. The molecular forms of MMP-9 were confirmed with specific anti-antibodies for MMP-9 (Calbiochem, Merck KGaA, Darmstadt, Germany) by the western blot method. The total level of MMP-9 was defined as the combined gelatinolytic activity of actMMP-9 and proMMP-9. The percentual expression of the activated form of MMP-9 (Act% of MMP-9) based on densitometric evaluation was calculated as actMMP‐9/(proMMP‐9 + actMMP‐9) [20].

### 2.5. Statistical Analysis

Normality of the measured variables was visually inspected. Associations with continuous patient characteristics, as well as interactions between the variables, were assessed using Spearman's rank correlation, while the relation to categorical patient characteristics was analyzed using the Mann-Whitney *U* test or the Kruskal-Wallis test. The Bonferroni correction was applied in multiple comparisons, and *P* values < 0.05 were considered significant.

The predictive values of the studied variables were determined by binary logistic regression analysis. To facilitate the interpretation of the results, regression analyses were conducted using median-cut values of the variables. The odds ratios for death, death or severe neurological sequelae, and death or any neurological sequelae of all the studied molecules were adjusted for the level of consciousness on admission. This was done due to the pivotal impact of the child's presenting status on the outcomes of BM [21]. Local regression was applied to visually estimate the risk of death for different values of MMP-8.

Statistical analyses were conducted with IBM SPSS Statistics software, version 24 (IBM Corp., NY, US), except for the local regression procedure which was performed in R, version 3.4.2 (R Foundation for Statistical Computing, Vienna, Austria).

## 3. Results

### 3.1. Study Group

This series comprised 245 children with bacterial meningitis originating from the Dominican Republic (*n* = 104), Venezuela (*n* = 54), Paraguay (*n* = 51), and Ecuador (*n* = 36). Scarcity of CSF allowed the MMP-9 measurements from 240, MMP-8 from 236, and TIMP-1 and MPO from 232 patients. The patient characteristics, causative agents, and disease outcomes are summarized in Table 1.

### 3.2. Median Values and Associations with Baseline Patient Characteristics

**Figure 1:**
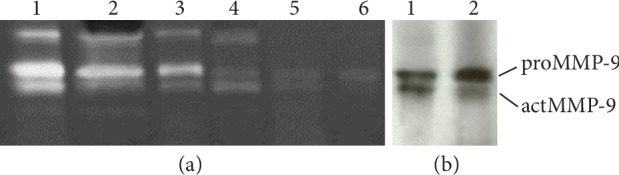
Gelatin zymography analysis of meningitis cerebrospinal fluid (CSF). (a) Lanes 1-3 represent CSF with elevated levels of MMP-9, and lanes 4-6 represent CSF with low levels of MMP-9. (b) Lanes 1 and 2 represent western immunoblot analysis of MMP-9. Mobilities of 92 kDa pro MMP-9 (proMMP-9) and active MMP-9 (actMMP-9) species are indicated on the right.

The expression rates in the CSF for MMP-8, TIMP-1, MPO, proform of MMP-9 (proMMP-9), and active form of MMP-9 (actMMP-9) were 99%, 94%, 84%, 93%, and 73%, respectively. The median concentrations were 453 ng/mL (interquartile range (IQR) 189–1,593 ng/mL) for MMP-8, 232 ng/mL (IQR 53–1,251) for TIMP-1, and 5,018 ng/mL (1,993–16,927) for MPO. The corresponding median densitometric values were 0.21 (IQR 0.00–0.78) for actMMP-9, 0.42 (IQR 0.14–1.46) for proMMP-9, and 0.64 (IQR 0.16–2.21) for total MMP-9 activity (Figure 1).

While comparing the CSF MMP levels with the baseline patient characteristics, some differences between MMP-9 and MMP-8 were noted. First, total MMP-9 correlated with the patient's presenting condition: the worse the GCS score, the higher the MMP-9 level (Table 2). In contrast, a similar association was not detected for MMP-8. Second, the MMP-8 concentrations correlated with the CSF white cell count, CSF protein, and CSF glucose levels, while no such correlations were found for total MMP-9 (Table 2). Finally, the duration of preadmission illness associated with the levels of total MMP-9, although not with MMP-8 (Table 2).

MPO correlated, similarly to total MMP-9, negatively with the GCS score on admission but was also positively associated with the CSF white cell count and protein level. In contrast, TIMP-1 associated inversely with these CSF parameters. The relation of TIMP-1 to the GCS score on admission mimicked that of total MMP-9 and MPO (Table 2). Moreover, TIMP-1 was the only variable relating to patient age: the younger the patient, the higher the CSF TIMP-1 concentration.

Of all the examined variables, only MMP-9 distinguished between the different causative agents (*P* = 0.006). *Streptococcus pneumoniae* meningitis induced the highest MMP-9 levels, although the only significant difference was found between meningitides caused by *S. pneumoniae* and *Haemophilus influenzae* (*P* = 0.001).

### 3.3. Associations between the Studied Molecules

A correlation matrix for the studied variables is presented in Table 3. MPO correlated positively with MMP-8, with the molar ratio of MMP-8 to TIMP-1, and with total MMP-9. In contrast, the higher the MPO concentration, the lower that of TIMP-1. The concentration of TIMP-1 related negatively to the percentage of active MMP-9; however, such a negative correlation was not noted with MMP-8 or with total MMP-9. High levels of MMP-8 related to higher total MMP-9, but not to a higher percentage of active MMP-9.

### 3.4. Relation to Disease Outcomes

**Figure 2:**
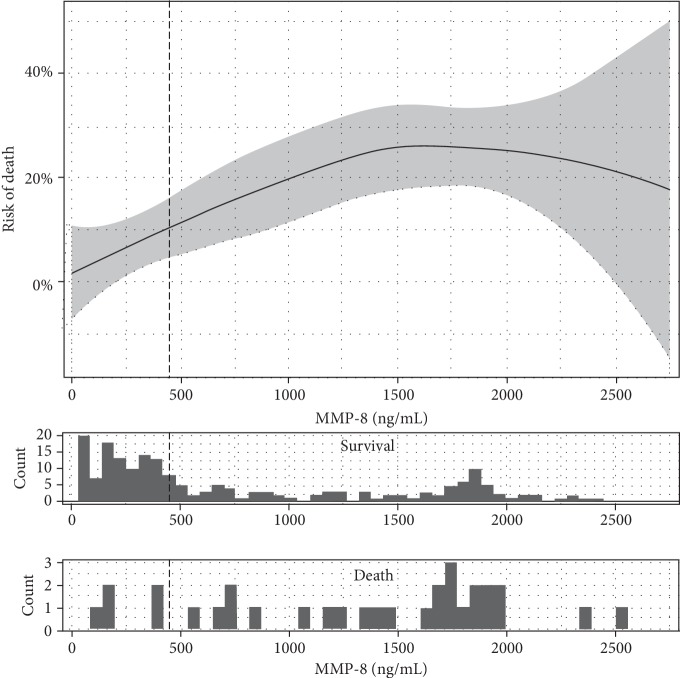
The risk of death estimated for different concentrations of MMP-8 using local regression. The vertical dashed line represents the median MMP-8 concentration, while the area shaded in grey shows the 95% confidence interval.

The crude and adjusted odds ratios (ORs) and 95% confidence intervals (CIs) for death and for the composite outcomes of death and severe or any neurological sequelae are presented in Tables 4(a)–4(c), respectively. Notably, MMP-8 emerged as the best predictor of demise; even when adjusted for the presenting condition, a CSF concentration above the median increased the odds of death almost fivefold (OR 4.9, 95% CI 1.8–12.9).  Figure 2 shows the risk of death versus different values of MMP-8. A less clear relationship was found between the molar ratio of MMP-8 to TIMP-1 and the odds of death (adjusted OR 2.8, 95% CI 1.2–7.0). None of the other variables was independent predictors of death after adjustment (Table 4(a)).

When neurological deficits were included in the calculations, the results remained similar. For death or severe neurological sequelae, the adjusted OR for median-cut MMP-8 was 2.3 (95% CI 1.2–4.6); for death or any neurological sequelae, the equivalent OR was 2.2 (95% CI 1.2–4.0) (Tables 4(b) and 4(c)).

A CSF MPO concentration above the median doubled the odds of death or severe neurological sequelae (OR 2.2, 95% CI 1.1–4.4). However, it did not increase the odds of death or any neurological sequelae. Adjusted median-cut values for CSF MMP-9, TIMP-1, or the molar ratio of MMP-8 and TIMP-1 did not predict worse composite outcomes (Tables 4(b) and 4(c)).

## 4. Discussion

Our results suggest that elevated MMP-8 levels in the CSF of children with BM predict poor disease outcomes, especially an increased risk of death. Furthermore, we demonstrated that the actions of MPO, MMP-8, and MMP-9 during the proinflammatory burst of BM run parallel to each other, counterbalanced by TIMP-1.

Prior data on these issues are sparse. A few studies have registered upregulation of MMP-8 in CSF [13, 14], but no association with disease outcomes has been reported [13]. The rather small sample sizes in those analyses probably concealed the findings we observed here, although also different statistical methods were applied.

Previous data suggest that MPO can oxidatively modify the function of MMP-8, MMP-9, and TIMP-1 [9–11, 22]. The positive association of MPO with MMP-8 and MMP-9 in our study might demonstrate such an interplay between these mediators or simply indicate that these enzymes share a common origin or a factor that leads to upregulation. Similarly, the negative relation of MPO to TIMP-1 could reflect the inactivation of TIMP-1 by MPO products [9], which would further strengthen the proinflammatory burden and favor the enhanced proteolytic actions of MMPs. Our results do not, however, show causality between MPO and the studied MMPs and TIMP-1 and thus leave the definitive relationship between these CSF inflammatory mediators unclear. Indeed, MPO and these MMPs are probably upregulated through similar pathways involving enhanced neutrophil degranulation during inflammation, while the expression of TIMP-1 seems substantially different [23].

The comparison of the MMP concentrations with the other CSF parameters revealed that MMP-8 related to the CSF white cell count, while MMP-9 did not. The contradictory results of previous studies suggest that CSF pleocytosis is merely one of several factors affecting the amount of MMPs in the CSF [12–14, 24]. As presynthetized and prepacked MMP-8 and MMP-9 are stored in different subcellular granules in neutrophils, their selective degranulation could partly account for the differences—certain conditions, proinflammatory mediators, and/or microbial virulence factors might regulate and promote the degranulation of a specific enzyme [22, 25].

MMP-8 and MMP-9 differed in terms of correlation with the patient's presenting condition. Consistent with our previous study [12], the lower the patient performed in the GCS, the higher the MMP-9 level in CSF. No such relation was detected for MMP-8. Considering that the regulation of these enzymes differs in terms of cellular gene expression and degranulation [6–8, 22], we reason that this difference might, at least partially, be explained by dynamics. In line with this, the duration of preadmission illness associated with CSF MMP-9, but not with MMP-8.

We acknowledge limitations in our study. Due to increased vaccination against *H. influenzae*, the etiology of BM in children has dramatically changed since the collection of the patient cohort. While the MMP-9 level indeed differed between the causative bacteria, no such differences were detected for MMP-8 or MPO, suggesting that other factors than bacterial etiology may be more important in regulating the expression of these enzymes. However, also the use of zymography as a semiquantitative method complicates the comparison of MMP-9 with the other inflammatory mediators. The CSF samples had been kept frozen for a long time before these analyses. Our data does not point towards degradation or inactivation of these molecules during storage, although this possibility cannot be fully excluded. Our main finding of MMP-8 predicting poor disease outcomes was, however, related to the median value of all samples and not to the absolute concentration in the CSF. Finally, our clinical study does not clarify the pathways triggered by MMP-8 that would explain for the higher risk of poor outcomes. Based on previous data, we hypothesize that both damaging the blood-brain barrier and enhancing the local inflammation contribute to this observation.

In conclusion, CSF MMP-8 presents as an attractive prognostic marker for BM in children. Our results, however, warrant a prospective study with a control group to validate these findings and to further elucidate the potential of this molecule, for example, in the differential diagnosis of meningitis. Rapid quantitative point-of-care tests for MMP-8 in salivary/body fluids are already available [26, 27].

## Figures and Tables

**Table 1:** Patient characteristics.

Characteristic	Result
Total number of patients	245
Male sex (%)	140/245 (57.1)
Age, median months (IQR)	8 (5–19)
Duration of preadmission illness, median days (IQR)	2 (1–3)
GCS score on admission, median (IQR)	13 (11–14)
Etiology, number of cases (%)	
*Haemophilus influenzae*	122 (49.8)
*Streptococcus pneumoniae*	64 (26.1)
*Neisseria meningitidis*	10 (4.1)
Other	12 (4.9)
Unknown	37 (15.1)
CSF test results on admission	
Leukocyte count, median cells/*μ*L (IQR)	2,500 (900–8,050)
Protein level, median mg/dL (IQR)	158 (94–257)
Glucose level, median mg/dL (IQR)	15 (6–32)
Disease outcomes, number of patients	
Death (%)	33/245 (13.5)
Death or severe neurological sequelae (%)	58/242*^a^* (24.0)
Death or any neurological sequelae (%)	112/241*^b^* (46.5)

IQR: interquartile range. *^a^*Information on severe neurological sequelae was missing for 3 patients. *^b^*Information on any neurological sequelae was missing for 4 patients.

**Table 2:** Associations between MMP-8, MPO, TIMP-1, and MMP-9 and baseline patient characteristics*^a^.*

Characteristic	MMP-8	MPO	TIMP-1	Total MMP-9
Age	*ρ* 0.071	*ρ* -0.013	**ρ** **-0.173**	*ρ* 0.009
	*P* = 0.28	*P* = 0.84	**P** = 0.009	*P* = 0.89
Duration of preadmission illness in days	*ρ* -0.057	*ρ* -0.182	*ρ* 0.161	**ρ** **0.242**
	*P* = 0.55	*P* = 0.05	*P* = 0.09	**P** = 0.008
GCS on admission	*ρ* -0.074	**ρ** **-0.179**	**ρ** **-0.135**	**ρ** **-0.258**
	*P* = 0.27	**P** = 0.007	**P** = 0.04	**P** < 0.001
CSF test results				
CSF white cell count	**ρ** **0.356**	**ρ** **0.238**	**ρ** **-0.275**	*ρ* -0.019
	**P** < 0.001	**P** = 0.001	**P** < 0.001	*P* = 0.79
CSF protein level	**ρ** **0.350**	**ρ** **0.440**	**ρ** **-0.189**	*ρ* 0.041
	**P** < 0.001	**P** < 0.001	**P** = 0.007	*P* = 0.56
CSF glucose	**ρ** **-0.219**	**ρ** **-0.198**	*ρ* -0.050	*ρ* 0.090
	**P** = 0.001	**P** = 0.004	*P* = 0.46	*P* = 0.18

*^a^*Associations were calculated using Spearman's rank correlation. Significant correlations are in bold.

**Table 3:** Correlation matrix for the studied molecules*^a^.*

Variable	MPO	MMP-8	TIMP-1	Total MMP-9	Act% of MMP-9	MMP8/TIMP-1 molar ratio
MPO	N/A	**ρ** **0.496**	**ρ** **-0.361**	**ρ** **0.153**	*ρ* 0.128	**ρ** **0.528**
		**P** < 0.001	**P** < 0.001	**P** = 0.02	*P* = 0.05	**P** < 0.001
MMP-8	**ρ** **0.496**	N/A	*ρ* -0.052	**ρ** **0.160**	*ρ* 0.051	**ρ** **0.556**
	**P** < 0.001		*P* = 0.43	**P** = 0.02	*P* = 0.44	**P** < 0.001
TIMP-1	**ρ** **-0.361**	*ρ* -0.052	N/A	*ρ* -0.052	**ρ** **-0.158**	**ρ** **-0.476**
	**P** < 0.001	*P* = 0.43		*P* = 0.44	**P** = 0.02	**P** < 0.001
Total MMP-9	**ρ** **0.153**	**ρ** **0.160**	*ρ* -0.052	N/A	**ρ** **0.570**	**ρ** **0.186**
	**P** = 0.02	**P** = 0.02	*P* = 0.44		**P** < 0.001	**P** = 0.005
Act% of MMP-9	*ρ* 0.128	*ρ* 0.051	**ρ** **-0.158**	**ρ** **0.570**	N/A	*ρ* 0.108
	*P* = 0.05	*P* = 0.44	**P** = 0.02	**P** < 0.001		*P* = 0.10
MMP8/TIMP-1 molar ratio	**ρ** **0.528**	**ρ** **0.556**	**ρ** **-0.476**	**ρ** **0.186**	*ρ* 0.108	N/A
	**P** < 0.001	**P** < 0.001	**P** < 0.001	**P** = 0.005	*P* = 0.10	

*^a^*Correlations were calculated using Spearman's rank correlation. Significant correlations are in bold. Act% of MMP-9 represents the percentual expression of the active form of MMP-9.

**Table:** (a) Odds ratios for death*^a^*

Variable	Unadjusted OR (95% CI)	*P* value	Adjusted OR*^b^* (95% CI)	*P* value
MMP-8	5.3 (2.1–13.4)	<0.001	4.9 (1.8–12.9)	0.001
MPO	2.8 (1.2–6.3)	0.02	2.0 (0.8–4.8)	0.12
Total MMP-9	2.2 (1.0–4.8)	0.04	1.4 (0.6–3.3)	0.41
TIMP-1	0.7 (0.3–1.5)	0.34	0.7 (0.3–1.6)	0.37
MMP-8/TIMP-1 molar ratio	3.3 (1.4–7.8)	0.005	2.8 (1.2–7.0)	0.02

*^a^*The results were obtained with binary logistic regression analysis. The ORs were calculated for cerebrospinal fluid concentrations above the median. *^b^*Adjusted for the clinical condition on admission.

**Table:** (b) Odds ratios for death or severe neurological sequelae*^a^*

Variable	Unadjusted OR (95% CI)	*P* value	Adjusted OR*^b^* (95% CI)	*P* value
MMP-8	2.5 (1.3–4.6)	0.005	2.3 (1.2–4.6)	0.02
MPO	2.8 (1.4–5.2)	0.002	2.2 (1.1–4.4)	0.03
Total MMP-9	1.5 (0.8–2.7)	0.21	0.9 (0.5–1.8)	0.78
TIMP-1	0.8 (0.4–1.5)	0.51	0.8 (0.4–1.5)	0.46
MMP-8/TIMP-1 molar ratio	1.5 (0.8–2.8)	0.20	1.3 (0.6–2.5)	0.50

*^a^*The results were obtained with binary logistic regression analysis. The ORs were calculated for cerebrospinal fluid concentrations above the median. *^b^*Adjusted for the clinical condition on admission.

**Table:** (c) Odds ratios for death or any neurological sequelae*^a^*

Variable	Unadjusted OR (95% CI)	*P* value	Adjusted OR*^b^* (95% CI)	*P* value
MMP-8	2.2 (1.3–3.8)	0.003	2.2 (1.2–4.0)	0.007
MPO	1.8 (1.1–3.1)	0.02	1.6 (0.9–2.8)	0.12
Total MMP-9	1.8 (1.1–3.0)	0.03	1.2 (0.7–2.2)	0.47
TIMP-1	1.4 (0.8–2.3)	0.24	1.2 (0.7–2.2)	0.45
MMP-8/TIMP-1 molar ratio	1.2 (0.7–2.0)	0.49	1.1 (0.6–2.0)	0.70

*^a^*The results were obtained with binary logistic regression analysis. The ORs were calculated for cerebrospinal fluid concentrations above the median. *^b^*Adjusted for the clinical condition on admission.

## Data Availability

The datasets analyzed during this study are not publicly available due to patient-related confidentiality. However, these data are available from the corresponding author on reasonable request.

## References

[1] GBD 2015 Child Mortality Collaborators (2016). Global, regional, national, and selected subnational levels of stillbirths, neonatal, infant, and under-5 mortality, 1980–2015: a systematic analysis for the global burden of disease study 2015. *The Lancet*.

[2] Koedel U., Klein M., Pfister H. W. (2010). New understandings on the pathophysiology of bacterial meningitis. *Current Opinion in Infectious Diseases*.

[3] Khokha R., Murthy A., Weiss A. (2013). Metalloproteinases and their natural inhibitors in inflammation and immunity. *Nature Reviews. Immunology*.

[4] Leppert D., Lindberg R. L., Kappos L., Leib S. L. (2001). Matrix metalloproteinases: multifunctional effectors of inflammation in multiple sclerosis and bacterial meningitis. *Brain Research Reviews*.

[5] Nissinen L., Kahari V. M. (2014). Matrix metalloproteinases in inflammation. *Biochimica et Biophysica Acta (BBA) - General SubjectsBiochimica et Biophysica Acta*.

[6] Yan C., Boyd D. D. (2007). Regulation of matrix metalloproteinase gene expression. *Journal of Cellular Physiology*.

[7] Loffek S., Schilling O., Franzke C. W. (2011). Series “matrix metalloproteinases in lung health and disease”: biological role of matrix metalloproteinases: a critical balance. *European Respiratory Journal*.

[8] Chakraborti S., Mandal M., Das S., Mandal A., Chakraborti T. (2003). Regulation of matrix metalloproteinases: an overview. *Molecular and Cellular Biochemistry*.

[9] Wang Y., Rosen H., Madtes D. K. (2007). Myeloperoxidase inactivates TIMP-1 by oxidizing its N-terminal cysteine residue: an oxidative mechanism for regulating proteolysis during inflammation. *Journal of Biological Chemistry*.

[10] Weiss S. J. (1989). Tissue destruction by neutrophils. *The New England Journal of Medicine*.

[11] Meli D. N., Christen S., Leib S. L. (2003). Matrix metalloproteinase–9 in pneumococcal meningitis: activation via an oxidative pathway. *The Journal of Infectious Diseases*.

[12] Roine I., Pelkonen T., Bernardino L. (2014). Predictive value of cerebrospinal fluid matrix metalloproteinase-9 and tissue inhibitor of metalloproteinase-1 concentrations in childhood bacterial meningitis. *The Pediatric Infectious Disease Journal*.

[13] Leppert D., Leib S. L., Grygar C., Miller K. M., Schaad U. B., Hollander G. A. (2000). Matrix metalloproteinase (MMP)-8 and MMP-9 in cerebrospinal fluid during bacterial meningitis: association with blood-brain barrier damage and neurological sequelae. *Clinical Infectious Diseases*.

[14] Lindberg R. L., Sorsa T., Tervahartiala T. (2006). Gelatinase B [matrix metalloproteinase (MMP)-9] and collagenases (MMP-8/-13) are upregulated in cerebrospinal fluid during aseptic and bacterial meningitis in children. *Neuropathology and Applied Neurobiology*.

[15] Peltola H., Roine I., Fernandez J. (2007). Adjuvant glycerol and/or dexamethasone to improve the outcomes of childhood bacterial meningitis: a prospective, randomized, double-blind, placebo-controlled trial. *Clinical Infectious Diseases*.

[16] Myntti T., Rahkonen L., Nupponen I. (2017). Amniotic fluid infection in preterm pregnancies with intact membranes. *Disease Markers*.

[17] Visse R., Nagase H. (2003). Matrix metalloproteinases and tissue inhibitors of metalloproteinases: structure, function, and biochemistry. *Circulation Research*.

[18] Roine I., Pelkonen T., Lauhio A. (2015). Changes in MMP-9 and TIMP-1 concentrations in cerebrospinal fluid after 1 week of treatment of childhood bacterial meningitis. *Journal of Clinical Microbiology*.

[19] Sorsa T., Salo T., Koivunen E. (1997). Activation of type IV procollagenases by human tumor-associated trypsin-2. *Journal of Biological Chemistry*.

[20] Lahdentausta L., Leskelä J., Winkelmann A. (2018). Serum MMP-9 diagnostics, prognostics, and activation in acute coronary syndrome and its recurrence. *Journal of Cardiovascular Translational Research*.

[21] Roine I., Peltola H., Fernandez J. (2008). Influence of admission findings on death and neurological outcome from childhood bacterial meningitis. *Clinical Infectious Diseases*.

[22] Sorsa T., Tjäderhane L., Konttinen Y. T. (2006). Matrix metalloproteinases: contribution to pathogenesis, diagnosis and treatment of periodontal inflammation. *Annals of Medicine*.

[23] Vandooren J., Swinnen W., Ugarte-Berzal E. (2017). Endotoxemia shifts neutrophils with TIMP-free gelatinase B/MMP-9 from bone marrow to the periphery and induces systematic upregulation of TIMP-1. *Haematologica*.

[24] Yushchenko M., Weber F., Mäder M. (2000). Matrix metalloproteinase-9 (MMP-9) in human cerebrospinal fluid (CSF): elevated levels are primarily related to CSF cell count. *Journal of Neuroimmunology*.

[25] Ding Y., Haapasalo M., Kerosuo E., Lounatmaa K., Kotiranta A., Sorsa T. (1997). Release and activation of human neutrophil matrix metallo- and serine proteinases during phagocytosis of *fusobacterium nucleatum, porphyromonas gingivalis and treponema denticola*. *Journal of Clinical Periodontology*.

[26] Sorsa T., Gursoy U. K., Nwhator S. (2016). Analysis of matrix metalloproteinases, especially MMP-8, in gingival crevicular fluid, mouthrinse and saliva for monitoring periodontal diseases. *Periodontology 2000*.

[27] Sorsa T., Gieselmann D., Arweiler N. B., Hernandez M. (2017). A quantitative point-of-care test for periodontal and dental peri-implant diseases. *Nature Reviews Disease Primers*.

